# Impact of Hemoglobin and Iron Deficiency on Mortality in Patients with Acute Myocardial Infarction in Intensive Care Units: A Retrospective Study from MIMIC-IV

**DOI:** 10.31083/RCM28261

**Published:** 2025-05-13

**Authors:** Fangyuan Luo, Zhe Wang, Tong Gao, Baofu Wang, Yijie Gao, Mengru Liu, Hong Jiang, Xianlun Li

**Affiliations:** ^1^China-Japan Friendship Hospital (Institute of Clinical Medical Sciences), Chinese Academy of Medical Sciences & Peking Union Medical College, 100029 Beijing, China; ^2^Department of Integrative Medicine Cardiology, China-Japan Friendship Hospital, 100029 Beijing, China; ^3^Department of Cardiology, Beijing Anzhen Hospital, Capital Medical University, 100029 Beijing, China

**Keywords:** intensive care units, hemoglobins, iron deficiencies, myocardial infarction, mortality

## Abstract

**Background:**

Anemia and iron deficiency (ID) are common in patients with acute myocardial infarction (AMI), especially those in intensive care units (ICU). This study investigated the impact of hemoglobin (Hb) and ID on the short-term mortality of critically ill patients with AMI.

**Methods:**

Overall 992 AMI patients with their first ICU admission were included in this analysis. ID was defined as serum ferritin <100 ng/mL or transferrin saturation (TSAT) <20%. Patients were categorized into four groups according to their Hb concentrations and the presence of ID. Kaplan-Meier survival analysis was used to assess differences in all-cause mortality between the different groups, and Cox regression models to identify risk factors for all-cause mortality.

**Results:**

Anemia was present in 89.5% of patients, while 65.9% suffered from ID. Patients in the group with Hb <9 g/dL and without ID were the youngest, yet they exhibited the highest severity scores. The Kaplan–Meier analysis showed that this group had a higher rate of all-cause mortality compared to the other three groups (Log-rank test *p = *0.005). Moreover, multivariate Cox regression analysis revealed that Hb <9 g/dL and no ID was associated with a higher risk of all-cause mortality at 120 days (hazard ratio 1.512, 95% confidence interval 1.031–2.217, *p = *0.034) when compared to the reference group (Hb ≥9 g/dL and no ID). Additionally, multivariate Cox regression analysis showed that lower Hb was linked to increased rates of all-cause mortality at 30, 60, 90, and 120 days. Elevated levels of ferritin and TSAT were also associated with increased all-cause mortality at 60, 90, and 120 days. Compared to patients without ID, those with ID had a decreased risk of all-cause mortality at 60, 90, and 120 days.

**Conclusions:**

Anemia and ID were prevalent in ICU patients with AMI. Patients with Hb <9 g/dL and without ID showed higher 120-day all-cause mortality. Additionally, lower Hb, elevated ferritin, and increased TSAT levels were identified as significant risk factors for short-term all-cause mortality in these patients.

## 1. Introduction

Anemia is prevalent in hospitalized patients with acute myocardial infarction (AMI), Previous study indicates that anemia is present in up to one in four patients with acute coronary syndrome (ACS) [1]. In the realm of critical care, anemia is exceedingly prevalent, affecting approximately two-thirds of patients upon admission [2]. The presence of anemia can further reduce the oxygen supply to ischemic myocardial tissue caused by ACS. Existing study indicates that patients with ACS who also have anemia are at a heightened risk for more severe outcomes, including significantly higher rates of in-hospital and long-term mortality [3]. Furthermore, these patients are more likely to experience heart failure and have an elevated risk of major bleeding events, and reinfarction [3, 4, 5].

Beyond anemia, iron deficiency (ID), defined by reduced iron bioavailability and storage, is a key factor influencing oxygen metabolism. It is prevalent among patients with cardiovascular disease, affecting nearly half of those with coronary artery disease (CAD) [6]. Regardless of the presence of anemia, ID has been identified as a critical predictor of adverse outcomes in heart failure (HF) patients [7]. A study has demonstrated that intravenous iron replacement can significantly improve the prognosis for these patients [8]. However, the impact of ID on the prognosis of patients with AMI remains controversial. Moreover, there is a notable scarcity of research on its correlation with short-term outcomes.

Anemia and ID may have additional associations with adverse outcomes in patients with AMI. Currently, there is a lack of research on the impact of hemoglobin (Hb) concentration and ID on the prognosis of severe patients with AMI. Therefore, this study utilized the American Medical Information Mart for Intensive Care IV (MIMIC-IV) cohort to focus on critically ill patients with AMI and explore the effects of Hb and ID on their short-term prognosis.

## 2. Methods

### 2.1 Study Population

**Fig. 1.**
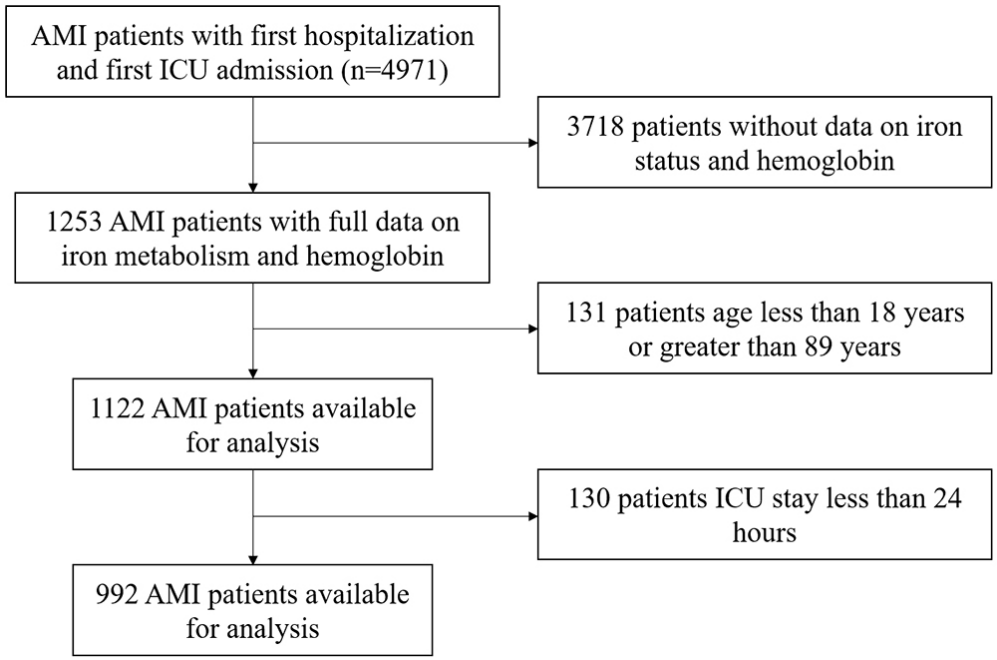
**The flowchart illustrated the selection of patients from the MIMIC-IV database**. AMI, acute myocardial infarction; ICU, intensive care units; MIMIC-IV, Medical Information Mart for Intensive Care IV.

This retrospective study used the freely accessible MIMIC-IV database (https://mimic.mit.edu), which contains over 50,000 intensive care units (ICU) admissions at Beth Israel Deaconess Medical Center (Boston, Massachusetts) from 2008 to 2019 [9]. The population of this study is critically ill patients diagnosed with AMI in the MIMIC-IV database. The international classification of diseases (ICD) codes used to screen for the diagnosis of AMI mainly include 410 in ICD-9, and I21 in ICD-10. Inclusion criteria were: (1) First hospital admission with initial ICU stay; (2) Diagnosis includes AMI. Exclusion criteria were: (1) Age outside the range of 18 to 89 years; (2) ICU stay less than 24 hours; (3) Missing data for key research indicators (Hb, ferritin, serum iron, total iron binding capacity). The population screening process can be found in Fig. 1.

### 2.2 Data Collection

We have undertaken a comprehensive training course offered by the National Institutes of Health and successfully passed the examination on “Protecting Human Research Participants” (certification number: 60366313). Navicat for PostgreSQL (version 16.0, PremiumSoft CyberTech Ltd., Hong Kong, China) was employed to filter data and extract baseline information, comorbidities, vital signs, laboratory tests, disease severity scoring, treatment measures conducted during hospitalization, and follow-up outcomes. The diagnosis of complications is based on ICD-9 and ICD-10 codes. The indicators related to iron status (ferritin, serum iron, total iron binding capacity) were based on the first measurement results during the patient’s hospitalization. Hb and other laboratory tests, as well as vital signs, are all based on the average values within 24 hours after the patient’s admission to the ICU.

### 2.3 Outcome and Definitions

The study’s primary endpoint was 120-day mortality after admission. Secondary endpoints were 30-day, 60-day, and 90-day mortality. Anemia was defined according to the World Health Organization criteria as Hb <13 g/dL in men and <12 g/dL in women. Transferrin saturation (TSAT) = serum iron concentration/total iron binding capacity×100. ID was defined as serum ferritin <100 ng/mL or TSAT <20%. Patients were also diagnosed with ID when ferritin ≥100 ng/mL and TSAT <20% [10]. We categorize patients into four groups based on Hb concentration and whether they are iron deficient. The groups are as follows: Hb ≥9 g/dL and no ID, Hb ≥9 g/dL and ID, Hb <9 g/dL and no ID, and Hb <9 g/dL and ID.

### 2.4 Statistical Analysis

Continuous data were presented as mean ± standard deviation or median (interquartile range, IQR), while categorical data were expressed in terms of frequency and percentage. For four-group comparisons of continuous variables, the analysis of variance (ANOVA) or Kruskal-Wallis test was used according to whether the variables obeyed normal distribution and had homoscedastic variance; the chi-square test or Fisher’s exact test was employed for comparing categorical variables. The Kaplan-Meier curves and the log-rank test were utilized to estimate and compare all-cause death between the study groups. Univariate and multivariate Cox regression analyses were performed to evaluate the risk of all-cause death. To account for potential confounding, variables with *p* < 0.05 from the univariate analyses, along with important mortality-related clinical factors were substituted into the multivariate analyses. Hazard ratio (HR) and corresponding 95% confidence interval (CI) were reported. Moreover, an analysis using restricted cubic spline (RCS) was performed to identify any possible nonlinear associations between the Hb, TSAT, Log-ferritin, and all-cause mortality after adjusting confounders that were statistically significant in univariate analyses. A two-sided *p* < 0.05 was considered statistically significant. All statistical analyses were conducted by SPSS software (version 25.0, IBM Corporation, Armonk, NY, USA) and R (version 4.1.3, R Foundation for Statistical Computing, Vienna, Austria).

## 3. Results

### 3.1 Patient Characteristics

A total of 992 critically ill patients with AMI were included in the analysis following screening for appropriate criteria. The Hb concentration of the overall study population was 9.8 ± 1.9 g/dL, with 888 (89.5%) patients having anemia, 364 (36.6%) patients having Hb <9 g/dL, and 654 (65.9%) patients experiencing ID. These patients were categorized into four groups based on Hb concentration and whether they were ID. Overall, 210 patients (21.2%) had Hb ≥9 g/dL and no ID, 418 patients (42.1%) had Hb ≥9 g/dL and ID, 128 patients (12.9%) had Hb <9 g/dL and no ID, and 236 patients (23.8%) had Hb <9 g/dL and ID. The baseline characteristics of these 992 patients are outlined in Table 1.

**Table 1.** **Baseline characteristics**.

	All (n = 992)	Hb ≥9 g/dL and no ID (n = 210)	Hb ≥9 g/dL and ID (n = 418)	Hb <9 g/dL and no ID (n = 128)	Hb <9 g/dL and ID (n = 236)	*p*-value
Age (years)	72.4 (63.8, 79.6)	73.1 (64.5, 80.0)	71.9 (62.5, 78.8)	69.4 (61.5, 77.7)	75.6 (67.6, 80.8)	<0.001
Female (n, %)	408 (41.1)	82 (39.0)	178 (42.6)	44 (34.4)	104 (44.1)	0.262
Hypertension (n, %)	782 (78.8)	156 (74.3)	327 (78.2)	101 (78.9)	198 (83.9)	0.097
Congestive heart failure (n, %)	644 (64.9)	119 (56.7)	270 (64.6)	77 (60.2)	178 (75.4)	<0.001
Atrial fibrillation (n, %)	361 (36.3)	75 (35.7)	150 (35.9)	38 (29.7)	98 (41.5)	0.154
Dyslipidemia (n, %)	530 (53.4)	107 (51.0)	229 (54.8)	63 (49.2)	131 (55.5)	0.542
Diabetes (n, %)	512 (51.6)	91 (43.3)	214 (51.2)	68 (53.1)	139 (58.9)	0.012
Cerebrovascular disease (n, %)	137 (13.8)	21 (10.0)	66 (15.8)	17 (13.3)	33 (14.0)	0.264
Chronic pulmonary disease (n, %)	309 (311)	66 (31.4)	131 (31.3)	26 (20.3)	86 (36.4)	0.018
Chronic kidney diseases (n, %)	453 (45.7)	80 (38.1)	168 (40.2)	76 (59.4)	129 (54.7)	<0.001
Heart rate (bpm)	84.3 ± 15.4	83.3 ± 14.7	85.0 ± 16.4	85.6 ± 15.6	83.4 ± 13.7	0.328
SBP (mmHg)	115.7 ± 15.7	116.0 ± 15.7	114.8 ± 14.8	117.6 ± 18.5	115.9 ± 15.5	0.330
RR (cpm)	19.9 ± 3.7	19.4 ± 3.7	20.2 ± 3.9	20.1 ± 3.5	19.7 ± 3.4	0.068
T (°C)	36.8 ± 0.6	36.8 ± 0.6	36.8 ± 0.6	36.8 ± 0.5	36.8 ± 0.6	0.654
WBC (K/µL)	11.7 (8.8, 15.2)	11.8 (8.8, 15.7)	11.8 (9.2, 15.4)	10.6 (7.2, 15.1)	11.2 (8.6, 14.7)	0.209
Platelets (K/µL)	198.5 (143.6, 265.3)	182.8 (136.5, 247.3)	204.3 (155.0, 267.6)	175.3 (103.8, 232.4)	213.8 (151.0, 294.0)	<0.001
Troponin_T (µg/L)	0.24 (0.08, 0.70)	0.20 (0.06, 0.59)	0.25 (0.08, 0.83)	0.20 (0.08, 0.57)	0.27 (0.08, 0.68)	0.250
BUN (mmol/L)	31.7 (19.0, 52.0)	27.5 (17.9, 44.5)	27.8 (17.5, 45.1)	47.3 (30.0, 72.0)	38.3 (22.1, 61.0)	<0.001
Cr (mg/dL)	1.5 (1.0, 2.6)	1.4 (0.9, 2.7)	1.3 (1.0, 2.3)	2.3 (1.2, 4.0)	1.6 (1.1, 2.5)	<0.001
FBG (mg/dL)	144.5 (117.4, 186.9)	142.8 (117.1, 183.8)	146.2 (119.6, 190.8)	136.6 (116.5, 180.3)	146.1 (117.6, 187.6)	0.560
Sodium (mmol/L)	138.0 ± 4.9	138.2 ± 6.0	137.9 ± 4.6	138.1 ± 4.5	138.2 ± 4.3	0.797
Potassium (mmol/L)	4.4 ± 0.7	4.4 ± 0.7	4.4 ± 0.7	4.6 ± 0.8	4.5 ± 0.7	0.014
Calcium (mg/dL)	8.4 ± 0.8	8.3 ± 0.8	8.5 ± 0.8	8.3 ± 0.8	8.3 ± 0.7	0.002
Anion gap (mEq/L)	16.3 ± 4.4	16.1 ± 4.5	16.4 ± 4.4	17.6 ± 4.7	15.7 ± 3.8	0.001
INR	1.3 (1.2, 1.5)	1.3 (1.1, 1.5)	1.3 (1.2, 1.5)	1.4 (1.2, 1.6)	1.4 (1.2, 1.6)	0.004
PT (seconds)	14.2 (12.7, 16.8)	13.9 (12.4, 16.1)	14.1 (12.5, 16.5)	14.7 (13.0, 18.0)	14.6 (13.0, 17.5)	0.009
PTT (seconds)	36.8 (29.6, 58.3)	37.1 (29.8, 61.6)	37.7 (29.6, 60.8)	35.4 (29.6, 52.6)	35.7 (29.6, 56.7)	0.781
SOFA	6.0 ± 3.6	6.4 ± 3.9	5.7 ± 3.5	7.2 ± 3.6	5.7 ± 3.3	<0.001
SAPS 2	42.3 ± 13.5	43.2 ± 13.5	41.1 ± 13.8	45.6 ± 14.3	41.9 ± 12.2	0.009
SAPS 3	52.3 ± 20.4	53.0 ± 22.7	51.1 ± 20.4	58.4 ± 21.3	50.6 ± 17.2	0.002
LODS	5.8 ± 3.0	5.7 ± 3.2	5.7 ± 3.1	6.7 ± 3.0	5.8 ± 2.7	0.007
OASIS	33.6 ± 8.6	34.5 ± 8.8	33.5 ± 8.8	34.5 ± 8.8	32.6 ± 8.1	0.075
SIRS	3.0 (2.0, 3.0)	3.0 (2.0, 3.0)	3.0 (2.0, 3.0)	3.0 (2.0, 3.0)	3.0 (2.0, 3.0)	0.702
PCI (n, %)	129 (13.0)	23 (11.0)	71 (17.0)	9 (7.0)	26 (11.0)	0.009
CABG (n, %)	127 (12.8)	28 (13.3)	57 (13.6)	5 (3.9)	37 (15.7)	0.011
Mechanical ventilation (n, %)	896 (90.3)	185 (88.1)	386 (92.3)	112 (95.3)	213 (90.3)	0.229
RRT (n, %)	97 (9.7)	25 (11.9)	34 (8.1)	23 (18.0)	15 (6.4)	0.002
IABP (n, %)	61 (6.1)	10 (4.8)	38 (9.1)	4 (3.1)	9 (3.8)	0.011
Aspirin (n, %)	888 (89.5)	185 (88.1)	385 (92.1)	98 (76.6)	220 (93.2)	<0.001
Digoxin (n, %)	62 (6.3)	10 (4.8)	34 (8.1)	6 (4.7)	12 (5.1)	0.221
Diuretic (n, %)	778 (78.4)	146 (69.5)	332 (79.4)	100 (78.1)	200 (84.7)	0.001
LOS Hos (day)	11.6 (7.1, 18.5)	10.9 (6.2, 20.3)	11.0 (6.9, 18.5)	13.8 (8.6, 21.7)	12.1 (6.9, 17.0)	0.043
LOS ICU (day)	3.1 (1.9, 6.1)	3.1 (2.0, 6.4)	3.3 (2.0, 6.2)	3.1 (1.8, 5.8)	3.0 (1.9, 5.6)	0.363
In-hospital mortality (n, %)	163 (16.4)	33 (15.7)	62 (14.8)	30 (23.4)	38 (16.1)	0.142
7-day mortality (n, %)	65 (6.5)	16 (7.6)	25 (6.0)	7 (5.5)	17 (7.2)	0.796
30-day mortality (n, %)	179 (18.0)	37 (17.6)	65 (15.6)	34 (26.6)	43 (18.2)	0.045
60-day mortality (n, %)	236 (23.8)	52 (24.8)	81 (19.4)	46 (35.9)	57 (24.2)	0.002
90-day mortality (n, %)	266 (26.8)	57 (27.1)	93 (22.2)	51 (39.8)	65 (27.5)	0.001
120-day mortality (n, %)	289 (29.1)	60 (28.6)	108 (25.8)	54 (42.2)	67 (28.4)	0.005

**Abbreviations**: Hb, hemoglobin; ID, iron deficiency; SBP, systolic blood pressure; RR, respiratory rate; T, temperature; WBC, white blood cell count; BUN, blood urea nitrogen; Cr, creatinine; FBG, fasting blood glucose; IABP, intra-aortic balloon pump; INR, international normalized ratio; PT, prothrombin time; PTT, partial thromboplastin time; PCI, percutaneous coronary intervention; CABG, coronary artery bypass grafting; RRT, renal replacement therapy; SOFA, Sequential Organ Failure Assessment; SAPS, Simplified Acute Physiology Score; LODS, Logistic Organ Dysfunction Score; OASIS, Oxford Acute Severity of Illness Score; SIRS, Systemic Inflammatory Response Syndrome Score; LOS Hos, length of hospital stay.

Significant differences in age were observed among the groups (*p* < 0.001), with patients in the Hb <9 g/dL and no ID group being the youngest at 69.4 years and those in the Hb <9 g/dL and ID group being the oldest at 75.6 years. Despite being the youngest, patients in the Hb <9 g/dL and no ID group exhibited the highest severity scores (Sequential Organ Failure Assessment, Simplified Acute Physiology Score, Logistic Organ Dysfunction Score). Additionally, significant differences among the groups were noted in terms of comorbidities (congestive heart failure, diabetes, chronic pulmonary disease, chronic kidney disease), laboratory tests (platelets count, troponin T, blood urea nitrogen, creatinine, potassium, calcium, anion gap, international normalized ratio, prothrombin time), therapeutic measures (percutaneous coronary intervention, coronary artery bypass grafting, renal replacement therapy, intra-aortic balloon pump, aspirin, and diuretic), and mortality rates (30-day mortality, 60-day mortality, 90-day mortality, and 120-day mortality).

### 3.2 Association of Hemoglobin and Iron Deficiency With Clinical Outcomes

The overall mortality rate of critically ill patients with AMI was high, with an all-cause mortality rate of 29.1% at 120-days. No significant differences in the in-hospital and 7-day mortality rates were observed between the four groups of patients. However, patients in the Hb <9 g/dL and no ID group had significantly higher mortality rates at 30 days (26.6%), 60 days (35.9%), 90 days (39.8%), and 120 days (42.2%) compared to the other three groups.

**Fig. 2.**
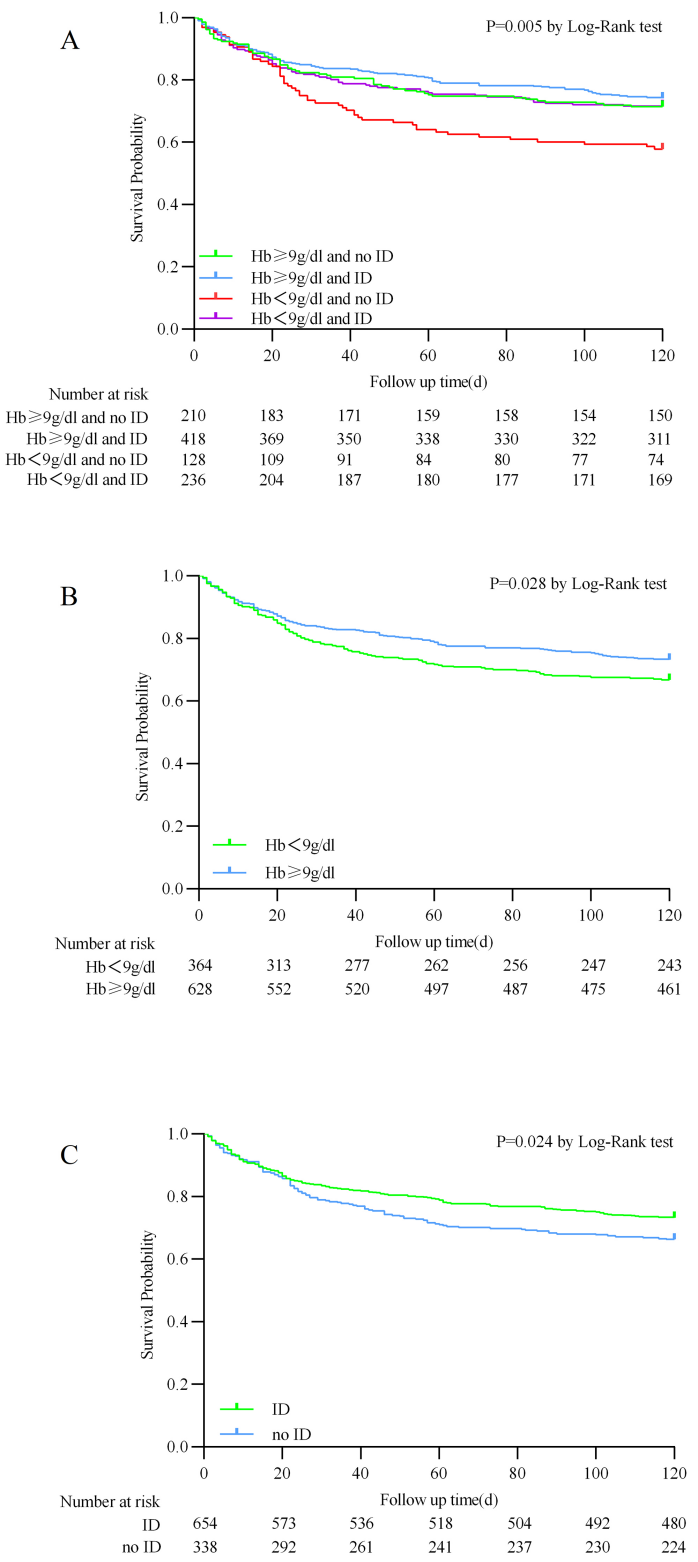
**Kaplan–Meier survival analysis curves for 120-day all-cause mortality**. (A) Stratified by hemoglobin and iron deficiency. (B) Stratified by hemoglobin. (C) Stratified by iron deficiency.

The Kaplan–Meier analysis showed that the Hb <9 g/dL and no ID group had a higher all-cause mortality rate at 120 days compared to the other three groups (log-rank *p* = 0.005). When categorizing patients solely based on Hb concentration, it was observed that patients with Hb <9 g/dL exhibit a markedly elevated all-cause mortality rate at the 120-day, in comparison to their counterparts with Hb ≥9 g/dL (log-rank *p* = 0.028). Furthermore, when patients were classified based on the absence or presence of ID, those without ID demonstrated a higher all-cause mortality rate at 120-days than patients with ID (log-rank *p* = 0.024, Fig. 2).

### 3.3 Risk Factors Associated With All-cause Mortality

In the univariate analysis, age, dyslipidemia, cerebrovascular disease, chronic pulmonary disease, heart rate, respiratory rate, systolic blood pressure, temperature, fasting blood glucose, blood urea nitrogen, anion gap, international normalized ratio, percutaneous coronary intervention (PCI), and coronary artery bypass grafting (CABG), and severity scores were significantly associated with the 120-day mortality rate (all *p* < 0.05). The results of the univariate analysis are detailed in **Supplementary Table 1**.

**Fig. 3.**
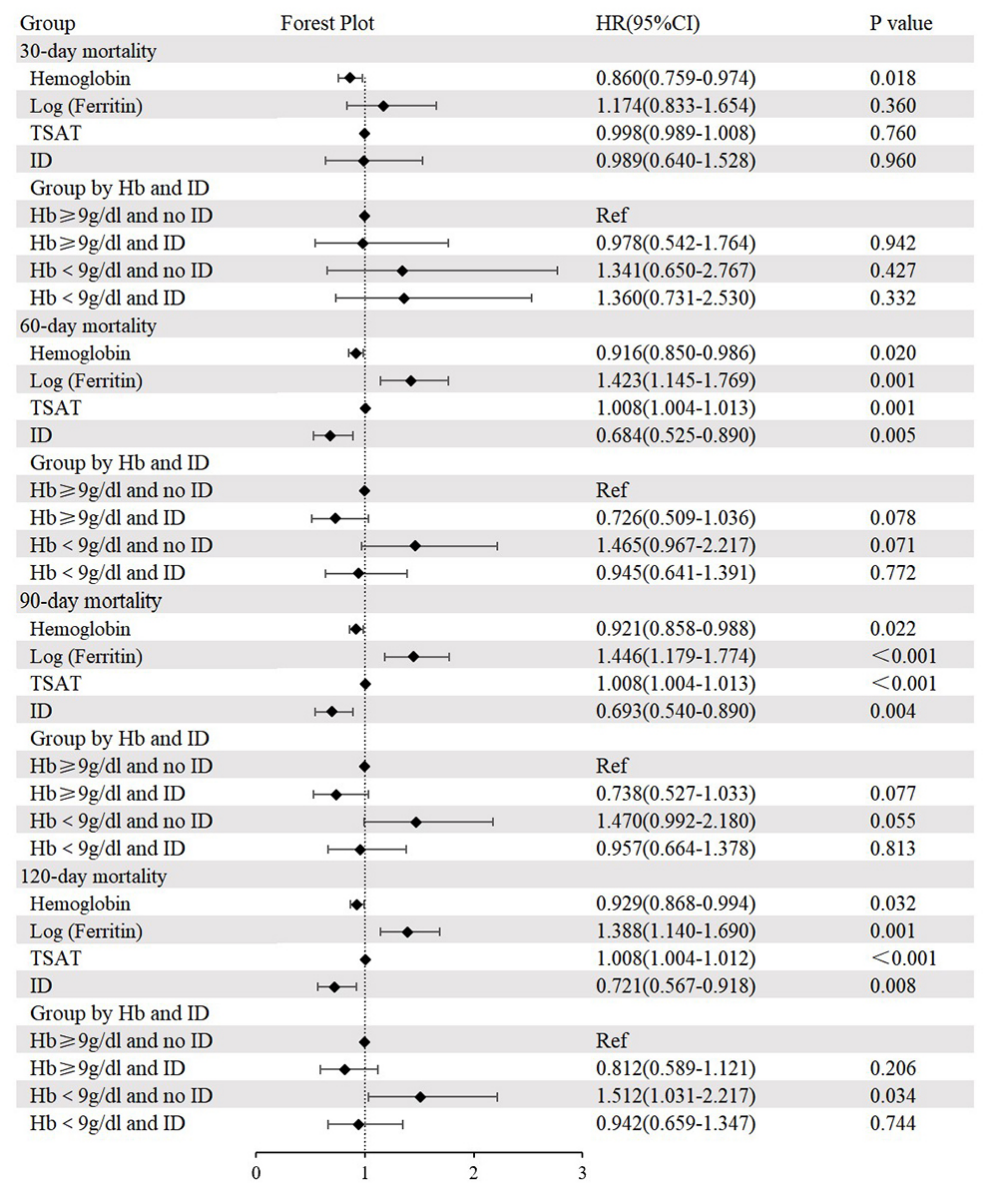
**Cox regression analysis and forest plot for 30-day, 60-day, 90-day, and 120-day all-cause mortality**. Adjusted for age, sex, dyslipidemia, cerebrovascular disease, chronic pulmonary disease, heart rate, respiratory rate, systolic blood pressure, temperature, fasting blood glucose, blood urea nitrogen, anion gap, international normalized ratio, percutaneous coronary intervention, and coronary artery bypass grafting. HR, hazard ratio; CI, confidence interval; TSAT, transferrin saturation; Ref, reference.

**Fig. 4.**
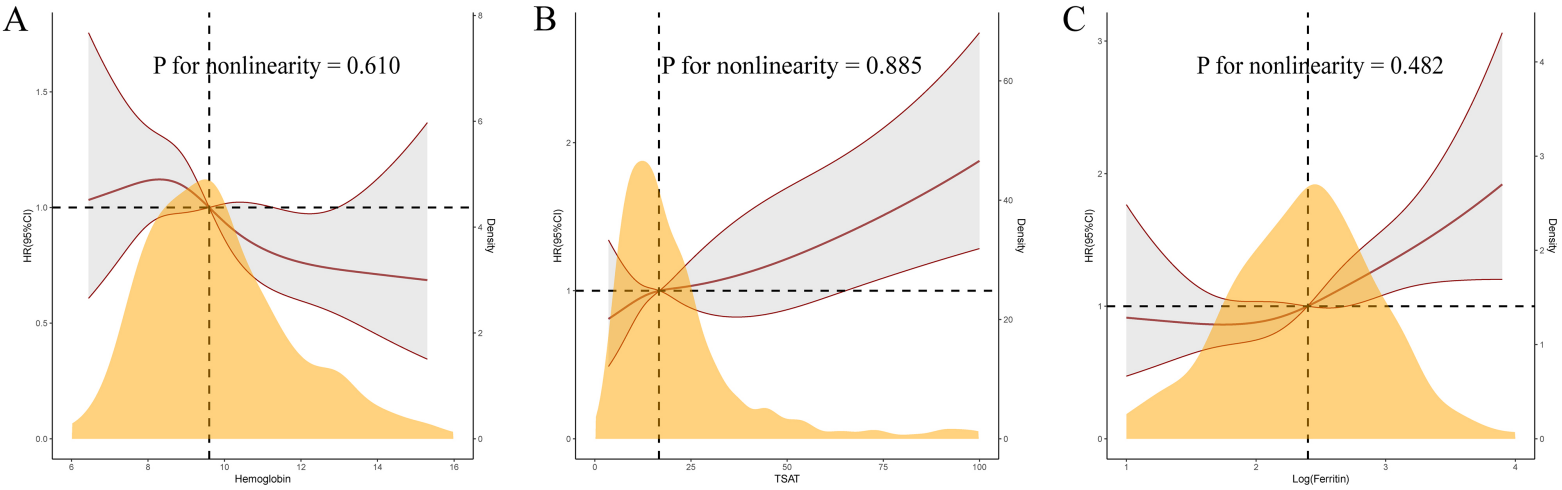
**Restricted cubic spline curves for 120-day mortality by hemoglobin (A), transferrin saturation (B), and log-ferritin (C) after covariates adjustment**.

Following adjustments for age, sex, dyslipidemia, cerebrovascular disease, chronic pulmonary disease, heart rate, respiratory rate, systolic blood pressure, temperature, fasting blood glucose, blood urea nitrogen, anion gap, international normalized ratio, PCI, and CABG, the multivariate Cox regression analysis revealed that the group with Hb <9 g/dL and no ID (HR 1.512, 95% CI 1.031–2.217, *p* = 0.034) was a risk factor for all-cause mortality at 120 days when compared to the reference group with Hb ≥9 g/dL and no ID (**Supplementary Table 2**). Simultaneously, increased Hb was associated with a lower all-cause mortality at 30, 60, 90, and 120 days. Elevated levels of Log-ferritin and TSAT were associated with increased all-cause mortality at 60, 90, and 120 days. The presence of ID was associated with a lower all-cause mortality at 60, 90, and 120 days (all *p* < 0.05, Fig. 3). The levels of Hb, TSAT, and log-ferritin were linearly related to the risk of all-cause mortality at 120-days according to the multivariable RCS model (all *p* for nonlinearity >0.05, Fig. 4).

### 3.4 Subgroup Analysis

**Fig. 5.**
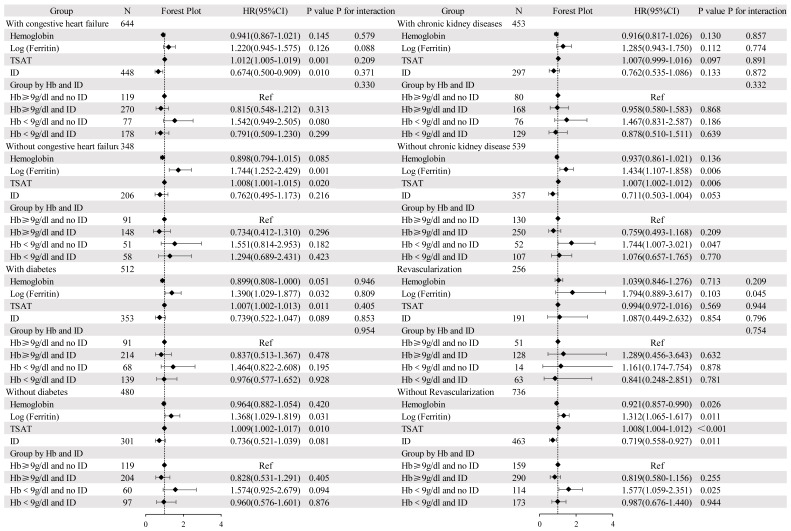
**Forest plots of hazard ratios for 120-day mortality in different subgroups**.

Following stratification for the presence of congestive heart failure, chronic kidney diseases, diabetes, and revascularization. An interaction was present between revascularization and log-ferritin (*p* = 0.045). Elevated log-ferritin (HR 1.312, 95% CI 1.065–1.617, *p* = 0.011) was associated with a higher mortality rate in patients who did not undergo revascularization, whereas this association was not statistically significant in patients who underwent revascularization. Log-ferritin levels were 2.41 ± 0.60 for patients without revascularization and 2.27 ± 0.58 for those with revascularization (*p* = 0.002). The remaining results of the subgroup analyses can be found in Fig. 5.

## 4. Discussion

This retrospective study leverages the MIMIC-IV database to investigate the impact of Hb concentration and ID on the short-term prognosis of critically ill patients with AMI. We observed several critical findings: Firstly, anemia and ID were common among patients with AMI in the ICU. Secondly, we identified that a decrease in Hb concentration, an increase in TSAT, and an elevation in log-ferritin were independent risk factors for short-term all-cause mortality in these patients. Unexpectedly, ID was associated with a better outcome. These relationships remain significant even after multi-factorial adjustment. Lastly, using patients with Hb ≥9 g/dL and no ID as the reference group, we found that those with Hb <9 g/dL and no ID exhibited a significantly higher risk of all-cause mortality within 120 days.

Anemia is prevalent among critically ill patients, impacting approximately two-thirds of those admitted [2]. The high prevalence of anemia, reaching 89.5% in our study population, can be attributed to the fact that the subjects are patients with AMI in the ICU, and iron status assessments may be more frequent in patients with anemia. Previous meta-analysis results showed that in patients with ACS, anemia was associated with a significantly increased risk of both early and late mortality [3]. In patients with ST-elevation myocardial infarction (STEMI), cardiovascular mortality increased as the Hb level fell below 14 g/dL [11]. The findings of our study align closely with these observations. The underlying mechanism may be attributed to the fact that anemia can exacerbate ischemia by reducing oxygen delivery to the compromised myocardium and increasing myocardial oxygen demand due to elevated cardiac output, which is necessary to maintain adequate systemic oxygen delivery [12, 13]. Furthermore, inflammation, which can cause anemia without the need for ID, and oxidative stress may also play significant roles in exacerbating adverse outcomes [14, 15].

Iron is an essential trace element that plays a crucial role in Hb synthesis, mitochondrial and cellular oxidative metabolism, the synthesis of essential biomolecules, and various other functions [16]. ID is linked with poorer quality of life, diminished exercise capacity, and worse prognosis in HF patients [17]. In a previous cohort study involving patients with HF, ID was typically defined as a serum ferritin level below 100 ng/mL or a serum ferritin level between 100 and 299 ng/mL when the TSAT is below 20%. These cutoff points were borrowed from the field of nephrology [18]. In a multicenter international study, a TSAT <20%, but not ferritin <100 ng/mL, was an independent predictor of mortality in patients with HF. Moreover, a low TSAT with ferritin >300 ng/mL represented patients with true ID but with high ferritin levels due to a marked inflammatory status [19]. Another study that compared different criteria for diagnosing ID in HF patients found that when ID was determined using current guidelines, it was not linked to adverse outcomes and lower serum ferritin levels were correlated with improved survival [20]. So, patients with TSAT <20% and ferritin >300 ng/mL were also considered to be ID in our study.

The impact of ID on the prognosis of patients with CAD remains controversial. In a retrospective subgroup analysis of patients with ACS from the AtheroGene cohort study, with a median follow-up of 4 years, ID was found to strongly predict cardiovascular mortality and non-fatal myocardial infarction [21]. Another previous research indicated that lower levels of TSAT were independently associated with an increased risk of long-term mortality in 252 elderly patients with ACS [22]. Fujinaga *et al*. [23] found that ID on admission was associated with elevated C-reactive protein (CRP) and advanced Killip stage, as well as increased in-hospital mortality after PCI in non-anemic patients with STEMI. However, another study involving 420 patients undergoing their first PCI for STEMI revealed that ID was associated with mitochondrial injury and with better in-hospital outcome [24]. Obradovic *et al*. [10], through an analysis of 427 patients with AMI complicated with cardiogenic shock in the Culprit Lesion Only PCI versus Multivessel PCI in Cardiogenic Shock (CULPRIT-SHOCK) trial, discovered that concomitant anemia without ID presence in patients was associated with an increased risk of all-cause mortality, renal replacement therapy, and the composite endpoint within 30 days post-hospitalization, but ID alone has no relevant impact on the clinical outcome. Similarly, our research concludes that Hb <9 g/dL and no ID exhibited a significantly increased risk of all-cause mortality within 120 days among patients with AMI in ICU when compared to the reference group with Hb ≥9 g/dL and no ID. Furthermore, the groups with Hb ≥9 g/dL and ID, as well as Hb <9 g/dL and ID, did not show a significant impact on clinical outcomes.

In this study, elevated levels of log-ferritin and TSAT were closely associated with poor prognoses, while ID corresponded with better outcomes. Several potential mechanisms are considered to underlie these associations. Primarily, the role of inflammation is critical. Serum ferritin, an acute-phase reactive protein, reflects systemic inflammatory states. Additionally, an elevation in ferritin might signify a complex interplay among ID, inflammation, and cellular damage [25]. This complexity means that elevated ferritin does not exclude the presence of ID. Therefore, the diagnostic utility of ferritin is limited in patients with coexisting conditions that have an inflammatory component. Furthermore, iron has been implicated in catalyzing the formation of reactive oxygen species, promoting the oxidation of lipoproteins, contributing to vascular dysfunction, and generating free radicals [26, 27]. Lastly, ferroptosis, an iron-dependent form of cell death, may play an important role. Previous studies have demonstrated that ferroptosis is involved in both the early and middle stages of myocardial infarction, as well as in myocardial ischemia-reperfusion injury [28, 29, 30].

This retrospective analysis using the MIMIC-IV database is the first to examine the impact of Hb and ID on the prognosis of ICU patients with AMI, yielding unexpected results that ID is linked to a better prognosis. The findings indicate that for critically ill patients with AMI, a population with a high mortality rate, Hb, ferritin, and TSAT can be valuable tools for risk stratification and management. While timely correction of low Hb concentration may theoretically enhance patient outcomes, it is important to recognize that such treatments may also influence iron status. Therefore, a cautious approach is warranted when addressing Hb levels in this population, and further research is needed to explore the optimal strategies for managing Hb while considering the broader implications for iron status. While iron supplementation can benefit patients with heart failure and ID, such treatment in critically ill patients with AMI and ID should be more cautious, and its effectiveness remains to be confirmed. Whether the conclusions of this study apply to non-severe patients with AMI still needs further exploration, and the optimal diagnostic methods for ID in the AMI population require additional research.

### Limitations

Several limitations need to be addressed in this study. Firstly, this is a small-scale retrospective study that only included patients with both Hb and iron markers measured, potentially enriching the cohort with individuals already suspected of anemia and leading to selection bias, highlighting the need for larger prospective cohort studies to validate these findings. Secondly, the research focuses on patients in the ICU with AMI, and whether its conclusions apply to all AMI patients remains uncertain. Thirdly, the study’s data, based on public databases, have missing information, leading to the omission of some crucial indicators that may affect the outcomes. Fourthly, due to the retrospective nature of the study, we cannot conduct an in-depth analysis of the causes of anemia and ID in the patients, such as hemorrhagic diseases and renal disorders. Lastly, there is a lack of a clear and reliable diagnostic standard for ID in patients with AMI, and different diagnostic criteria can impact the results. Therefore, large cohort studies are needed to further confirm the optimal diagnosis of ID in patients with AMI.

## 5. Conclusions

In severe patients with AMI, anemia and ID were common. Hb <9 g/dL without ID was associated with an increased 120-day all-cause mortality rate. Additionally, reduced Hb concentration, elevated ferritin, and increased TSAT were identified as risk factors for short-term all-cause mortality in these patients.

## Data Availability

This study analyzed publicly available datasets from the MIMIC-IV database (https://mimic.mit.edu/).

## Abbreviations

ACS, Acute coronary syndrome; AMI, Acute myocardial infarction; BUN, Blood urea nitrogen; CABG, Coronary artery bypass grafting; CAD, Coronary artery disease; Cr, Creatinine; FBG, Fasting blood glucose; Hb, Hemoglobin; HR, Hazard ratio; IABP, Intra-aortic balloon pump; ICU, Intensive care units; ID, Iron deficiency; INR, International normalized ratio; PCI, Percutaneous coronary intervention; PT, Prothrombin time; PTT, Partial thromboplastin time; RCS, Restricted cubic spline; RR, Respiratory rate; RRT, Renal replacement therapy; SBP, Systolic blood pressure; STEMI, ST-elevation myocardial infarction; T, Temperature; TSAT, Transferrin saturation; WBC, White blood cell count.

## Supplementary Material

Supplementary material associated with this article can be found, in the online version, at https://doi.org/10.31083/RCM28261.
