# Supplementary material for: Impact of Hemoglobin and Iron Deficiency on Mortality in Patients with Acute Myocardial Infarction in Intensive Care Units: A Retrospective Study from MIMIC-IV
Source: Rev Cardiovasc Med. 2025 May 13;26(5):28261. doi: 10.31083/RCM28261 (PMC12135660; doi:10.31083/RCM28261)
Supplement: Supplementary file 1 [file 2153-8174-26-5-28261-s1.docx]

Supplementary Table 1. Univariable Cox regression hazard analyses for 120-day mortality.

|  | HR | 95% CI | *p* |
| --- | --- | --- | --- |
| Age (years) | 1.035 | 1.023–1.047 | <0.001 |
| Female | 0.809 | 0.637–1.028 | 0.083 |
| Hypertension | 0.895 | 0.680–1.178 | 0.428 |
| Congestive heart failure | 1.129 | 0.883–1.444 | 0.334 |
| Atrial fibrillation | 1.167 | 0.922–1.477 | 0.200 |
| Dyslipidemia | 0.712 | 0.565–0.897 | 0.004 |
| Diabetes | 0.981 | 0.779–1.235 | 0.868 |
| Cerebrovascular disease | 1.532 | 1.139–2.059 | 0.005 |
| Chronic pulmonary disease | 1.337 | 1.052–1.699 | 0.018 |
| Chronic renal failure | 1.109 | 0.881–1.397 | 0.379 |
| HR (bpm) | 1.008 | 1.000–1.016 | 0.038 |
| SBP (mmHg) | 0.983 | 0.975–0.991 | <0.001 |
| RR (cpm) | 1.090 | 1.059–1.122 | <0.001 |
| T (°C) | 0.776 | 0.638–0.945 | 0.011 |
| WBC (K/μL) | 1.005 | 0.998–1.012 | 0.200 |
| Platelets (K/μL) | 0.999 | 0.998–1.001 | 0.297 |
| Troponin T (μg/L) | 1.012 | 0.965–1.062 | 0.619 |
| BUN (mmol/L) | 1.006 | 1.002–1.009 | 0.002 |
| Cr (mg/dL) | 1.002 | 0.955–1.051 | 0.929 |
| FBG (mg/dL) | 1.003 | 1.001–1.005 | 0.003 |
| Sodium (mmol/L) | 1.013 | 0.987–1.039 | 0.321 |
| Potassium (mmol/L) | 1.070 | 0.905–1.266 | 0.428 |
| Calcium (mg/dL) | 0.948 | 0.811–1.108 | 0.502 |
| Anion gap (mEq/L) | 1.059 | 1.034–1.085 | <0.001 |
| INR | 1.254 | 1.109–1.419 | <0.001 |
| PT (seconds) | 1.016 | 1.004–1.029 | 0.011 |
| PTT (seconds) | 1.006 | 1.001–1.010 | 0.010 |
| SOFA | 1.117 | 1.084–1.150 | <0.001 |
| SAPS 3 | 1.023 | 1.018–1.027 | <0.001 |
| SAPS 2 | 1.038 | 1.030–1.046 | <0.001 |
| LODS | 1.136 | 1.096–1.176 | <0.001 |
| OASIS | 1.052 | 1.039–1.065 | <0.001 |
| SIRS | 1.227 | 1.083–1.391 | 0.001 |
| PCI | 0.538 | 0.351–0.824 | 0.004 |
| CABG | 0.144 | 0.068–0.305 | <0.001 |
| Mechanical ventilation | 1.412 | 0.914–2.180 | 0.120 |
| RRT | 1.392 | 0.981–1.973 | 0.064 |
| IABP | 0.687 | 0.394–1.199 | 0.187 |
| Aspirin | 0.730 | 0.517–1.031 | 0.074 |
| Digoxin | 1.106 | 0.702–1.743 | 0.663 |
| Diuretic | 0.825 | 0.629–1.081 | 0.163 |

**Abbreviations:** Hb, hemoglobin; ID, iron deficiency; HR, heart rate; SBP, systolic blood pressure; RR, respiratory rate; T, temperature; WBC, white blood cell count; BUN, blood urea nitrogen; Cr, creatinine; FBG, fasting blood glucose; IABP, intra-aortic balloon pump; INR, international normalized ratio; PT, prothrombin time; PTT, partial thromboplastin time; PCI, percutaneous coronary intervention; CABG, coronary artery bypass grafting; RRT, renal replacement therapy; SOFA, Sequential Organ Failure Assessment; SAPS, Simplified Acute Physiology Score; LODS, Logistic Organ Dysfunction Score; OASIS, Oxford Acute Severity of Illness Score; SIRS, Systemic Inflammatory Response Syndrome Score.

Supplementary Table 2. Multivariable Cox regression hazard analyses for 120-day mortality.

|  | Unadjusted model | | Model1 | | Model2 | |
| --- | --- | --- | --- | --- | --- | --- |
|  | HR (95% CI) | *p* | HR (95% CI) | *p* | HR (95% CI) | *p* |
| Hb (continuous) | 0.931(0.875–0.991) | 0.024 | 0.952(0.893–1.015) | 0.130 | 0.929(0.868–0.994) | 0.032 |
| Hb＜9g/dL (categorical) | 1.297(1.027–1.639) | 0.029 | 1.246(0.986–1.575) | 0.065 | 1.299(1.018–1.657) | 0.035 |
| ID (categorical) | 0.763(0.602–0.966) | 0.024 | 0.748(0.591–0.948) | 0.016 | 0.721(0.567–0.918) | 0.008 |
| Ferritin (continuous) | 1.000(1.000–1.000) | 0.904 | 1.000(1.000–1.000) | 0.570 | 1.000(1.000–1.000) | 0.905 |
| Log-ferritin | 1.391(1.148–1.684) | 0.001 | 1.485(1.217–1.812) | <0.001 | 1.388(1.140–1.690) | 0.001 |
| TSAT (continuous) | 1.008(1.004–1.012) | <0.001 | 1.010(1.005–1.014) | <0.001 | 1.008(1.004–1.012) | <0.001 |
| Group by Hb and ID |  |  |  |  |  |  |
| Hb≥9g/dL and no ID | Ref |  | Ref |  | Ref |  |
| Hb≥9g/dL and ID | 0.883(0.644–1.210) | 0.439 | 0.923(0.673–1.266) | 0.619 | 0.812(0.589–1.121) | 0.206 |
| Hb＜9g/dL and no ID | 1.583(1.096–2.286) | 0.014 | 1.717(1.187–2.484) | 0.004 | 1.512(1.031–2.217) | 0.034 |
| Hb＜9g/dL and ID | 0.998(0.705–1.415) | 0.993 | 0.944(0.666–1.338) | 0.747 | 0.942(0.659–1.347) | 0.744 |

Note: Model1 adjusted for Age, Sex;

Model2 adjusted for Age, Sex, Dyslipidemia, Cerebrovascular disease, Chronic pulmonary disease, HR, SAP, RR, T, BUN, FBG, Anion gap, INR, PCI, CABG
